# Automatic implantable cardioverter defibrillator pocket infection due to *Providencia rettgeri*: a case report

**DOI:** 10.4076/1757-1626-2-8607

**Published:** 2009-08-24

**Authors:** Jorge Manuel Marull, Maria Elena De Benedetti

**Affiliations:** 1 Department of Internal Medicine, St Luke’s-Roosevelt Hospital Center-Columbia University College of Physicians and Surgeons 1000 10^th^ Avenue, New York, NY, 10019USA; 2 Department of Internal Medicine, St Luke’s-Roosevelt Hospital Center-Columbia University College of Physicians and Surgeons1000 10^th^ Avenue, New York, NY, 10019USA

## Abstract

Coagulase-negative staphylococci and *Staphylococcus aureus* are the commonest pathogens involved in infections of pacemaker-defibrillator systems. Among causative Gram-negative bacteria, infections due to *Klebsiella, Serratia, Pseudomonas, Acinetobacter* and other species have been reported. We report herein a unique case of an automatic implantable cardioverter defibrillator infection due to *Providencia rettgeri* in a 65-year-old male who was admitted to our service with bacteremia and infection of the generator and subcutaneous array in a recently implanted device.

## Introduction

Insertion of an automatic implantable cardioverter-defibrillator (ICD) reduces the occurrence of sudden death in patients at risk for ventricular tachycardia or ventricular fibrillation [1]. Despite the advent of transvenously placed devices and improvements in surgical techniques, device infection is still a serious complication due to associated morbidity, mortality and financial costs [2]. Management of this clinical entity is challenging since no current guidelines are available for optimal treatment. We present a case of an ICD pocket infection due to an uncommon pathogen.

## Case presentation

This is a case of a 65-year-old Jewish male who presented to our hospital complaining of a two-week history of sero-sanguineous oozing coming from a healing surgical wound overlying an ICD that had been implanted sixteen days prior to the day of admission at a different medical center. No purulent discharge was seen. The area became increasingly painful. The patient denied any fevers, chills, palpitations or other cardiovascular symptoms on admission. Vital signs were as follows: Temperature of 98.4°F, Blood pressure of 105/64 mmHg, Heart rate of 60 beats per minute, Respiratory rate of 14 breaths per minute and Oxygen saturation of 98% on room air. Physical examination revealed an eight-centimeter irregularly oval, warm, swollen, erythematous and tender area on the left anterior-superior chest wall. No local ulcers or new heart murmurs were noticed. Poor dentition, together with the presence of disheveling appearance was appreciated.

Past medical history was positive for non-ischemic dilated cardiomyopathy, congestive heart failure (NYHA class III), atrial fibrillation, essential hypertension and a recent syncopal episode. The patient had no history of alcohol, tobacco or illegal drug abuse. He was a retired messenger since 2004 and lived in a shelter. No family history of cardiomyopathy or oncologic diseases was noted.

Admission laboratory findings revealed mild leukocytosis together with mild normocytic normochromic anemia (Table 1). An electrocardiogram showed atrial paced rhythm at 60 beats per minute, a QRS complex of 102 msec and a QTc interval of 480 sec, no signs of ischemia were recorded. His home medications included Warfarin, Carvedilol, Lisinopril and Simvastatin.

**Table 1.** Hematologic and blood chemistry laboratory data

**Hematology**		**Chemistry**			
White cell count	10.5	Sodium	138	Protein	6.2
Hemoglobin	12.1	Potassium	4.6	Albumin	3.3
Hematocrit	34.8	Chloride	105	Total Bilirrubin	0.5
Platelet	231	BUN	23	AST	40
PTT	56	Creatinine	1.2	ALT	49
Prothromin time	29.7	Glucose	89	Alk Phosp	43
INR	2.9	C Reactive protein	1.6		

Ref: Partial thromboplastin time (PTT), International Normal Ratio (INR), Blood Urea Nitrogen (BUN), Aspartate aminotransferase (AST), Alanine aminotransferase (ALT), Alkaline Phosphatase (Alk Phos).

*Providencia rettgeri* grew in two sets of deep wound cultures, one set of superficial wound cultures, one set of peripheral blood cultures obtained on the day of admission as well as from the culture of the removed device. The antibiotic resistance pattern was similar in all the cultures (Table 2). A trans-esophageal echocardiography did not reveal vegetations or signs of endocarditis.

**Table 2.** Resistance profile of organism isolate

Amikacin	<=16	S	Ciprofloxacin	<=1	S
Amoxicillin/clavulanate	>16/8	R	Gentamicin	<=4	S
Ampicillin	>16	R	Imipenem	<=4	S
Ampicillin/sulbactam	16/8	I	Levofloxacin	<=2	S
Cefazolin	>16	R	Moxifloxacin	<=2	S
Cefepime	30	S	Piperacillin/tazobactam	<=16	S
Cefoxitin	<=8	S	Ticarcillin/clavulanate	<=16	S
Ceftazidime	<=8	S	Tobramycin	<=4	S
Ceftriaxone	<=8	S	Trimethoprim/Sulfa	>2/38	R

Ref: Susceptible (S), Intermediate (I), Resistant (R).

The entire implanted system, including the cardiac leads of a dual chamber cardioverter-defibrillator, Medtronic®, model D224DRG, Secura DR was removed on day two of admission. The wound was left opened, and dry to wet dressings were applied locally. Resolution of the infection was seen after the patient received a four-week course of intravenous Cefepime two grams every twelve hours, subsequent surveillance blood cultures revealed no growth and second intention healing of the surgical wound ensued. An external defibrillator vest was initially placed and as soon as the antibiotic regimen was completed a new dual chamber cardioverter-defibrillator was placed on the right side of the thoracic wall. The patient was subsequently discharged with adequate follow-up.

## Discussion

*Providencia rettgeri* (PR) has been frequently associated with colonization of indwelling urinary catheters and urinary tract infections, bacteremias, skin infections, traveler’s diarrhea, etc [3,4]. To the best of our knowledge, this microorganism has never been implicated in infections of automatic ICD, being this case the first one ever reported in the literature as evidenced by a MEDLINE database search. Axillae, human feces, urine, throat and perineum could serve as potential reservoirs for Providencia species, mainly in chronic debilitated patients. Exogenous sources include sewage contaminated natural waters [5]. A probable key feature in the pathogenesis of this patient’s infection was the formation of a biofilm composed mainly of host adhesins (fibrinogen, fibronectin, collagen) and bacteria that may have occurred soon after the insertion of the ICD [6]. The skin of our patient could have been colonized with PR before the adherence of the bacterium to the device since no foley catheter insertion had been reported on prior admissions.

PR is a facultative anaerobe, Gram-negative, motile, opportunistic bacterium that belongs to the genus Providencia, tribe Proteeae (along with the genus Proteus and Morganella), Enterobacteriaceae family. PR was first isolated by Rettger in 1904 during an epidemic of fowl cholera, but it was only studied in detail later in 1918 when it was called Bacterium rettgerei [7]. In 1951, Kauffmann applied the term Providencia to the genus described by Stuart et al. at Brown University in Providence, Rhode Island [8]. Many taxonomic changes of genus Providencia have taken place since then, which included a frequent overlap between the Gram-negative genera belonging to the tribe Proteeae. Currently, the genus Providencia includes five species: *P. stuartii*, *P. rettgeri*, *P. alcalifacens*, *P. rustigianii* and *P. heimbachae*. Providencia stuartii has been described as the commonest isolate in residents of nursing homes diagnosed with bacteremia [9]. In a recent epidemiologic study in a Canadian population, the incidence of isolates for the genus Providencia was found to be 3.4⁄100 000⁄year, with more infections reported as people aged and were residing in nursing homes [10].

Nosocomial outbreaks of highly resistant strains of PR causing urinary tract infections and bacteremias have been described. Epidemiologic factors included the intensive concomitant use of antibiotics and indwelling urinary catheters; while the use of gloves by personnel showed to be an effective control measure [11,12]. Our patient was kept on contact isolation until the end of the admission with no further cases reported on the same medical ward.

Resistant Enterobacteriaceae is a recent major health hazard. The transmission of resistance is frequently acquired through plasmids from other Gram-negative rods and it has been associated with the increasing use of antibiotics. The frequent isolation of extended spectrum beta-lactamase in *Providencia species* alarms about the risk of future nosocomial epidemics caused by this multi-resistant organism and is a considered an emerging problem [13,14]. The natural antibiotic susceptibility of PR includes numerous beta-lactam, fluoroquinolone and aminoglycoside antibiotics and a less resistant pattern than that of *P. stuartii* [15].

## Conclusion

Infections by PR and other Gram-negative bacteria should be considered when approaching a recent hospitalized patient with an early automatic ICD infection, and initial empiric antibiotic coverage for this bacterium may be warranted. The election of the antibiotic should be guided by culture susceptibilities given the increasing evidence of multi-drug resistant strains.

## Abbreviations

ICD: Automatic implantable cardioverter-defibrillator
NYHA: New York Heart Association
PR: *Providencia rettgeri*
P. stuartii: Providencia stuartii
